# Biological Actions, Implications, and Cautions of Statins Therapy in COVID-19

**DOI:** 10.3389/fnut.2022.927092

**Published:** 2022-06-22

**Authors:** Chengyu Liu, Wanyao Yan, Jiajian Shi, Shun Wang, Anlin Peng, Yuchen Chen, Kun Huang

**Affiliations:** ^1^Department of Transfusion Medicine, Wuhan Hospital of Traditional Chinese and Western Medicine, Tongji Medical College, Huazhong University of Science and Technology, Wuhan, China; ^2^Department of Pharmacy, Wuhan Fourth Hospital, Wuhan, China; ^3^Tongji School of Pharmacy, Tongji Medical College, Huazhong University of Science and Technology, Wuhan, China; ^4^Wuhan Third Hospital, Tongren Hospital of Wuhan University, Wuhan, China; ^5^Tongji-Rongcheng Center for Biomedicine, Huazhong University of Science and Technology, Wuhan, China

**Keywords:** SARS-CoV-2, COVID-19, statins, obesity, dyslipidemia, inflammation, immune response, thrombosis

## Abstract

The Coronavirus Disease 2019 (COVID-19) showed worse prognosis and higher mortality in individuals with obesity. Dyslipidemia is a major link between obesity and COVID-19 severity. Statins as the most common lipid regulating drugs have shown favorable effects in various pathophysiological states. Importantly, accumulating observational studies have suggested that statin use is associated with reduced risk of progressing to severe illness and in-hospital death in COVID-19 patients. Possible explanations underlie these protective impacts include their abilities of reducing cholesterol, suppressing viral entry and replication, anti-inflammation and immunomodulatory effects, as well as anti-thrombosis and anti-oxidative properties. Despite these benefits, statin therapies have side effects that should be considered, such as elevated creatinine kinase, liver enzyme and serum glucose levels, which are already elevated in severe COVID-19. Concerns are also raised whether statins interfere with the efficacy of COVID-19 vaccines. Randomized controlled trials are being conducted worldwide to confirm the values of statin use for COVID-19 treatment. Generally, the results suggest no necessity to discontinue statin use, and no evidence suggesting interference between statins and COVID-19 vaccines. However, concomitant administration of statins and COVID-19 antiviral drug Paxlovid may increase statin exposure and the risk of adverse effects, because most statins are metabolized mainly through CYP3A4 which is potently inhibited by ritonavir, a major component of Paxlovid. Therefore, more clinical/preclinical studies are still warranted to understand the benefits, harms and mechanisms of statin use in the context of COVID-19.

## Introduction

**Figure 1:**
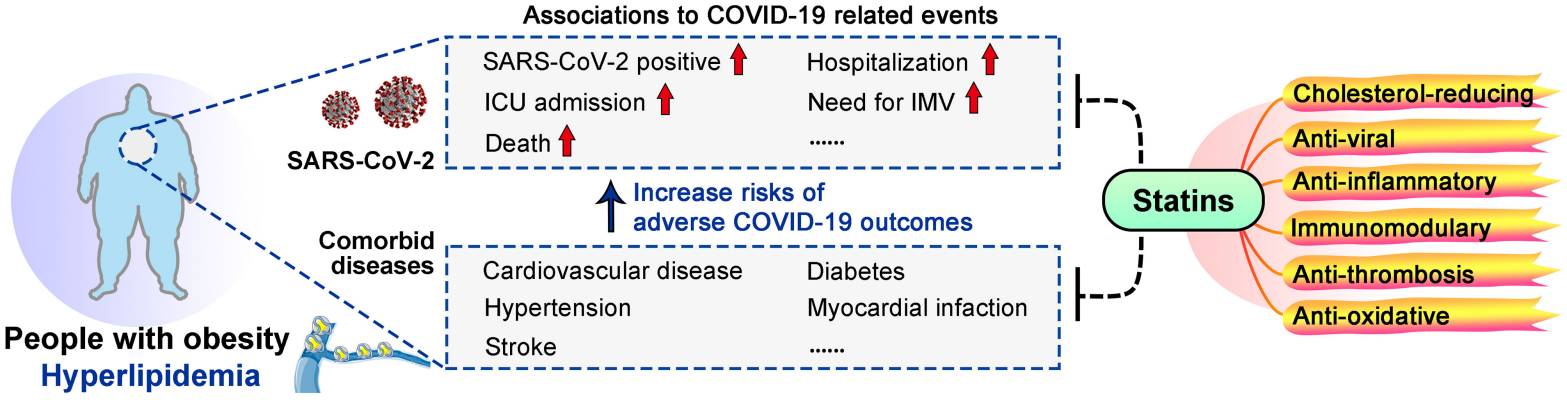
Links between obesity, COVID-19 and statins. Obesity increased the risks of adverse COVID-19 outcomes, and dyslipidemia is a major link between obesity and COVID-19 severity. Statins may benefit COVID-19 patients, especially those with obesity and dyslipidemia, due to their multiple effects.

Obese individuals are more vulnerable to the SARS-CoV-2 caused Coronavirus Disease 2019 (COVID-19) (1–3). Obese people have ~46% higher risk for SARS-CoV-2 positive, ~74% increased odds for intense care unit (ICU) admission and ~48% increased risk in deaths (4). Severe obesity [body mass index (BMI) ≥35] was significantly associated with the need for invasive mechanical ventilation (IMV) (5), and was an independent predictor for intubation outcome (6). Moreover, with hyperlipidemia as a major link, obese individuals are prone to cardiovascular disease, hypertension, diabetes, myocardial infarction and stroke, which are among recognized risk factors for adverse COVID-19 outcomes (7–12) (Figure 1).

Prevalence of dyslipidemia was 18–39.7% as a comorbid condition in hospitalized COVID-19 patients (13–15). A population-based analysis on 61.4 million adult patients suggested that patients with hyperlipidemic state had 70% increased odds for catching COVID-19 (16). Moreover, COVID-19 patients may develop dyslipidemia that leads to life-threatening metabolic diseases and thrombotic complications (17–19), lipid-regulating agents are thus considered for possible therapeutic effects against COVID-19.

Statins are the most commonly used lipid-regulating drugs, 145.8 million people used statins in 2018 (20). Statins are HMG-CoA reductase inhibitors that can reduce serum total cholesterol, low-density lipoprotein cholesterol (LDL-C) and triglyceride levels, and have other pleiotropic effects such as modulating immune response and alleviating inflammation (21–23). Structurally, statins are classified as lipophilic (atorvastatin, lovastatin, simvastatin, and fluvastatin) and hydrophilic (pravastatin and pitavastatin), while rosuvastatin has an intermediate behavior (24). Statin prescription is majorly under consideration for primary preventions of cardiovascular disease, and other pathologies such as thrombosis (25, 26). Rational use of different types of statins are recommended according to LDL-C lowering need, pre-existing cardiovascular events or related risk factors including dyslipidemia, diabetes, hypertension, and age (25, 26).

Observational studies have suggested protective effects of statins in COVID-19 patients. Statin use was associated with a 55% decreased risk for IMV (27), 22–30% reduced risk of ICU admission (28), and 30–47% lower risk for death (28–33) (Table 1). Moreover, statin use prior to admission was associated with 71% reduction in the odds of developing severe COVID-19 (34) and 73% for death (31). High-intensity statin use reduced the risk of death by 49% in COVID-19 patients with coronary artery disease patients (32) (Table 1). Possible explanations for these benefits of statins include their recognized cholesterol-reducing, anti-inflammatory and immunomodulatory capacities (21, 23, 49), and also their anti-viral, anti-thrombosis and anti-oxidative abilities (50–53) (Figure 1). However, possible side effects of statin should be considered, such as elevated creatine kinase (CK) and serum glucose levels, which are already elevated in severe COVID-19 (19, 54–56). Currently, clinical trials are being conducted worldwide to confirm the safety and benefits of statin use for COVID-19 patients, with criteria including mortality, thrombosis formation, need for ECMO or IMV, viral load etc. (Table 1).

**Table 1:** Associations between statins and COVID-19 outcomes, and clinical trials regarding statin use in COVID-19.

**References**	**Type of study**	**Study cohort**	**Key findings**	
**Associations between statins and COVID-19 outcomes**				
Song et al. (27)	Retrospective study	249 adult patients hospitalized with COVID-19 in Rhode Island, USA	After adjusting for age, sex, race, cardiovascular disease, chronic pulmonary disease, diabetes, and obesity, statin use was significantly associated with decreased risk for IMV [aOR = 0.45, (95% CI: 0.20–0.99)].	
Vahedian-Azimi et al. (28)	Meta-analysis	32,715 patients in 24 studies	Statin use is associated with significant reductions in ICU admission (OR = 0.78, 95% CI: 0.58–1.06; *n* = 10; *I*^2^ = 58.5%) and death (OR = 0.70, 95% CI: 0.55–0.88; *n* = 21; *I*^2^ = 82.5%) outcomes, with no significant effect on tracheal intubation (OR = 0.79; 95% CI: 0.57–1.11; *n* = 7; *I*^2^ = 89.0%). Death was reduced further by in-hospital application of stains (OR = 0.40, 95% CI: 0.22–0.73, *n* = 3; *I*^2^ = 82.5%), compared with pre-hospital use (OR = 0.77, 95% CI: 0.60–0.98, *n* = 18; *I*^2^ = 81.8%).	
Zhang et al. (29)	Retrospective study	13,981 cases of confirmed COVID-19 admitted in 21 hospitals from Hubei Province, China	The risk for 28-day all-cause mortality was 5.2 and 9.4% in the matched statin and non-statin groups, respectively, with an adjusted HR of 0.58; the use of statins in hospitalized subjects with COVID-19 was associated with a lower risk of all-cause mortality and a favorable recovery profile.	
Lee et al. (30)	Nested case-control study	10,448 COVID-19 patients who were hospitalized in Korea	Statins were prescribed in 533 (5.1%) patients. After adjusting for age, sex, and comorbidities, Cox regression showed a significant decrease in hazard ratio associated with the use of statins [aHR, 0.637 (95% CI, 0.425–0.953); *P* = 0.0283]. Statin use is correlated with lower mortality in COVID-19 patients.	
Memel et al. (31)	Cohort study	1,179 patients, 676 (57%) were male, 443 (37%) were >65 years old, and 493 (46%) had a BMI ≥30	Inpatient statin use reduced the hazard of death (HR, 0.566; *P* = 0.008). This association held among patients who did and those did not use statins before hospitalization [HR, 0.270 (*P* = 0.003) and 0.493 (*P* = 0.04), respectively]. Statin use was associated with improved time to death for patients aged >65 years but not for those ≤ 65 years old. Statin use during hospitalization for SARS-CoV-2 infection was associated with reduced 28-day mortality rates.	
Choi et al. (32)	Retrospective study	5,375 COVID-19 patients admitted to Mount Sinai Health System hospitals in New York	Compared to non-statin users, both low-to-moderate-intensity (aHR 0.62, 95% CI 0.51–0.76) and high-intensity statin users (aHR 0.53, 95% CI 0.43–0.65) had a reduced risk of death. Subgroup analysis of 723 coronary artery disease patients showed decreased mortality among high-intensity statin users compared to non-users (aHR 0.51, 95% CI 0.36–0.71). Statin use in patients hospitalized with COVID-19 was associated with a reduced in-hospital mortality. The protective effect of statin was greater in those with coronary artery disease.	
Rodriguez-Nava et al. (33)	Retrospective cohort study	87 adult patients with COVID-19 admitted to community hospital ICU in Evanston, IL, USA	In the multivariable Cox proportional hazards regression model, atorvastatin non-users had a 73% chance of faster progression to death compared with atorvastatin users (when probability = HR/HR + 1).	
Daniels et al. (34)	Retrospective single-center study	170 hospitalized patients with COVID-19 and 5,281 COVID-negative subjects at University of California San Diego Health	Statin use prior to admission was associated with reduced risk of severe COVID-19 (aOR 0.29, 95% CI 0.11–0.71, *P* < 0.01) and faster time to recovery among those without severe disease (aHR for recovery 2.69, 95% CI 1.36–5.33, *P* < 0.01). The association between statin use and severe disease was smaller in the COVID-negative cohort (*P* for interaction = 0.07).	
Rossi et al. (35)	Follow-up study	71 consecutive patients with a pre-existing chronic cardiovascular disease, who become ill from COVID-19	Among 42 statin users, 16/42 (38.1%) took a hydrophilic statin (rosuvastatin in 14 patients and pravastatin in 2), while 26/42 (61.9%) a lipophilic statin (atorvastatin in 22 patients, and simvastatin in 4). The group of lipophilic statins demonstrated a significant reduction in mortality respect both patients who do not take statins, and patients who assumed hydrophilic statins.	
Saeed et al. (36)	Observational study	4,252 patients (65 ± 16 years old; 47% female) were admitted with COVID-19, 37% (*n* = 1,570) were Hispanic	Patients with diabetes mellitus on a statin (*n* = 983) reduced cumulative in-hospital mortality (24 vs. 39%; *P* < 0.01) than those not on a statin (*n* = 1,283). Statin use in people with diabetes was associated with a reduced risk of in-hospital mortality during COVID-19. No difference in hospital mortality was noted in patients without diabetes mellitus on or off statin (20 vs. 21%; *P* = 0.82).	
De Spiegeleer et al. (37)	Retrospective study	154 COVID-19 diagnosed residents aged 86 ± 7 years in 2 Belgian nursing homes	Statin intake is associated with the absence of symptoms during COVID-19 (OR 2.91; 95% CI 1.27–6.71), which remained statistically significant after adjusting for covariates (aOR 2.65; 95% CI 1.13–6.68). In conclusion, statin intake in older, frail adults could be associated with a considerable beneficial effect on COVID-19 clinical symptoms.	
Lala et al. (38)	Retrospective study	2,736 patients with COVID-19 admitted to 1 of 5 Mount Sinai Health System hospitals in New York City	Statins have a protective effect and were associated with improved survival (HR 0.57, 95% CI 0.47–0.69).	
Gupta et al. (39)	Retrospective study	2,626 patients admitted with COVID-19, of whom 951 (36.2%) were antecedent statin users.	Among 1,296 patients (648 statin users, 648 non-statin users) identified with 1:1 propensity-score matching, statin use is significantly associated with lower odds of in-hospital mortality within 30 days in the propensity-matched cohort (OR 0.47, 95% CI 0.36–0.62, *P* < 0.001).	
Byttebier et al. (40)	Retrospective observational case-control study	959 COVID-19 patients admitted consecutively to four Belgian hospitals	Treatment with statins and ACEIs/ARBs reduced 28-day mortality in hospitalized COVID-19 patients. Moreover, combination treatment with these drugs resulted in a 3-fold reduction in the odds of hospital mortality (OR = 0.33; 95% CI 0.17–0.69). In-hospital treatment with statins, ACEIs/ARBs, and especially their combination saves lives.	
Ayeh et al. (41)	Retrospective study	4,447 patients hospitalized at the Johns Hopkins Hospital and affiliated hospitals with COVID-19, 594 (13.4%) were exposed to statins on admission.	The average treatment effect of statin use on COVID-19-related mortality was RR = 1.00 (95% CI: 0.99–1.01, *P* = 0.928), while its effect on severe COVID-19 infection was RR = 1.18 (95% CI: 1.11–1.27, *P* < 0.001).	
			Statin use was not associated with altered mortality, but with an 18% increased risk of severe COVID-19 infection.	
Kollias et al. (42)	Meta-analysis	41,807 patients, 14% with statin use	Statin therapy was associated with an about 35% decrease in the adjusted risk of mortality in hospitalized COVID-19 patients.	
Lee et al. (43)	Two independent population-based nationwide cohort studies	214,207 patients older than 20 years who underwent tests for SARS-CoV-2 infection in South Korea	Statin users were associated with a decreased likelihood of severe clinical outcomes [statin users, 3.98% (32/804); non-users, 5.40% (85/1,573); aRR 0.62; 95% CI 0.41–0.91] and length of hospital stay (statin users, 23.8 days; non-users, 26.3 days; adjusted mean difference −2.87; 95% CI −5.68 to −0.93) than non-users.	
			Prior statin use is related to a decreased risk of worsening clinical outcomes of COVID-19 and length of hospital stay but not to that of SARS-CoV-2 infection.	
Kow et al. (44)	Meta-analysis	8,990 COVID-19 patients in 4 studies	The pooled analysis revealed a significantly reduced hazard for fatal or severe disease with the use of statins (Pooled HR = 0.70; 95% CI 0.53–0.94) compared to non-use of statins in COVID-19 patients.	
Tan et al. (45)	Retrospective study	717 patients admitted to a tertiary center in Singapore for COVID-19 infection.	156 (21.8%) patients had dyslipidaemia and 97% of these were on statins. Logistic treatment models showed a lower chance of ICU admission for statin users when compared to non-statin users (Average treatment effect on statin (ATET): Coeff (risk difference): −0.12 (−0.23, −0.01); *P* = 0.028). Statin use was independently associated with lower ICU admission.	
**Study Title**	**Status**	**Locations**	**Summary**	**Key results**
**COVID-19 related clinical trials of statins**				
Intermediate-dose vs. standard prophylactic anticoagulation and statin vs. placebo in ICU patients with COVID-19 (NCT04486508)	Completed	Masih Daneshvari Hospital, Tehran, Iran, Islamic Republic of Iran	This study investigates the safety and efficacy of two pharmacological regimens on outcomes of critically-ill patients (Actual Enrollment: 600 participants) with COVID-19 using a 2 × 2 factorial design.	In adults with COVID-19 admitted to the ICU, atorvastatin was not associated with a significant reduction in the composite of venous or arterial thrombosis, treatment with extracorporeal membrane oxygenation, or all-cause mortality compared with placebo. The treatment was safe (46)
Effectiveness and safety of medical treatment for SARS-CoV-2 (COVID-19) in Colombia (NCT04359095)	Completed	6 hospitals in Colombia including Clinica santa Maria del lago, Clínica Reina Sofía, Fundacion Cardio Infantil, etc.	The study assesses the effectiveness and safety of rosuvastatin plus colchicine, emtricitabine/tenofovir, and their combined use in these patients. Six hundred and forty-nine patients agreed to participate and were enrolled in this study; among them, 633 (97.5%) were included in the analysis. The primary endpoint was 28-day all-cause mortality.	The combined use of emtricitabine with tenofovir disoproxil plus colchicine and rosuvastatin reduces the risk of 28-day mortality and the need for IMV in hospitalized patients with COVID-19 (47).
The impact of statin therapy in the COVID-19 patients (NCT05238402)	Completed	Deniz Demirci Antalya, Turkey	The study is retrospective single-center review of covid-19 patients (actual Enrollment: 707 participants). The study population was divided into patients who received a statin vs. those who did not receive a statin before the hospitalization. The primary outcome was in-hospital mortality during the follow-up period.	No results posted
Statin therapy and COVID-19 infection (NCT04407273)	Completed	Facultat de Medicina i Ciències de la Salut de Reus, Reus, Tarragona, Spain	This is a retrospective observational multicenter study. The SARS-CoV-2 severity of 2,159 COVID-19-infected patients with statin therapy was classified into 9 grades. Primary outcome is the WHO SARS-CoV-2 scale of severity (9 grades) achieved by COVID-19 patients, admitted in the hospital, with and without background statin therapy comparable in age and gender distribution.	No results posted
Randomized, embedded, multifactorial adaptive platform trial for community- acquired pneumonia (NCT02735707)	Recruiting	322 hospitals worldwide	The purpose of this study is to evaluate the effect of about 50 interventions, including statin use, to improve outcome of patients admitted to ICU with community-acquired pneumonia including COVID-19.	No results posted
Colchicine/statins for the prevention of COVID-19 complications (COLSTAT) trial (NCT04472611)	Recruiting	4 hospitals in United States including Bridgeport Hospital, Greenwich Hospital, Yale New Haven Hosptial System, Lawrence and Memorial Hospital	This is a randomized open-label study of the safety and efficacy of the combination of colchicine and Rosuvastatin in addition to standard of care (SOC) compared to SOC alone in hospitalized patients with SARS-CoV-2 (Estimated Enrollment: 466 participants). The primary endpoint is the 30-day composite of progression to severe COVID-19 disease.	No results posted
Managing endothelial dysfunction in critically ill COVID-19 patients at LAUMCRH (NCT04813471)	Recruiting	LAUMCRH Beirut, Lebanon	The study seeks to target endothelial dysfunction in critically ill patients with COVID-19 by giving them an endothelial protocol (L-arginine, Folic Acid, Statin, Nicorandil, Vitamin B complex) and monitor clinical outcome in those patients.	No results posted
Atorvastatin as adjunctive therapy in COVID-19 (NCT04380402)	Recruiting	Mount Auburn Hospital Cambridge, Massachusetts, United States	This study assesses whether adjunctive therapy of COVID-19 with atorvastatin reduces the deterioration in hospitalized patients and improves clinical outcome.	No results posted
Helping alleviate the longer-term consequences of COVID-19 (HEAL-COVID) (NCT04801940)	Recruiting	Addenbrookes Hospital, Cambridge, United Kingdom	HEAL-COVID aims to evaluate the impact of treatments on longer-term morbidity, mortality, re-hospitalization, symptom burden and quality of life associated with COVID-19. The first two treatment arms are Apixaban and Atorvastatin.	No results posted
Combination therapies to reduce carriage of SARS-CoV-2 and improve outcome of COVID-19 in ivory coast: a phase randomized IIb trial (NCT04466241)	Recruiting	2 hospitals in Côte D'Ivoire including Service des Maladies Infectieuses et Tropicales, Centre Hospitalier et Universitaire (CHU) and Treichville Abidjan, Côte D'Ivoire Center de Traitement des Maladies Infectieuses (CTMI)	This study proposes to study whether the combination of two drugs (These drugs include the LPV/r already in use in Côte d'Ivoire as well as an antihypertensive drug—telmisartan, and atorvastatin) is more effective than taking a single drug on reducing the viral load in the respiratory tract but also on reducing inflammation.	No results posted
Statin treatment for COVID-19 to optimize neurological recovery (NCT04904536)	Not yet recruiting	The George Institute for Global Health Sydney, New South Wales, Australia	This trial was designed to study whether atorvastatin treatment (40 mg/day) over 18 months can improve neurocognitive function in adults with long COVID neurological symptoms.	No results posted
A study of anticoagulation treatment patterns and outcomes of participants hospitalized with coronavirus disease 2019 (COVID-19) in Japan (NCT04828772)	Active, not recruiting	Medical Data Vision, Tokyo, Japan	This study plans to assess the benefits and harms of anticoagulants (including statins) vs. active comparator, placebo or no intervention in people hospitalized with COVID-19.	Compared with no treatment, anticoagulants may reduce all-cause mortality but the evidence comes from non-randomized studies and is very uncertain (48).
Managing endothelial dysfunction in COVID-19: a randomized controlled trial at LAUMC (NCT04631536)	Active, not recruiting	LAUMCRH Beirut, Lebanon	This trial will examine the potential therapeutic effect of a regiment composed of several medications including atorvastatin as adjunct to mainstream management, to further knowledge in treating COVID-19.	No results posted
Atorvastatin for reduction of 28-day mortality in COVID-19: RCT (NCT04952350)	Active, not recruiting	Mansoura University Hospitals Mansoura, Aldakahlia, Egypt	This randomized placebo-controlled double-blinded clinical trial aims to test the efficacy of administering atorvastatin 40 mg to hospitalized COVID-19 patients for 28 days on the all-cause 28-day mortality.	No results posted
Study of ruxolitinib plus simvastatin in the prevention and treatment of respiratory failure of COVID-19 (NCT04348695)	Unknown	Hospital Universitario Madrid Sanchinarro, Madrid, Spain	This project examines whether the combined use of ruxolitinib with simvastatin show a synergistic effect in the inhibition of viral entry and in the anti-inflammatory effect.	No results posted
Preventing cardiac complication of COVID-19 disease with early acute coronary syndrome therapy: a randomized controlled trial (NCT04333407)	Unknown	Charing Cross Hospital, London, United Kingdom	The trial plans to assess all-cause mortality 30 days after admission in COVID-19 patients (Estimated Enrollment: 3,170 participants) treated with different cardioprotective drugs, including Aspirin 75 mg, Clopidogrel 75 mg, Rivaroxaban 2.5 MG, Atorvastatin 40 mg, Omeprazole 20 mg.	No results posted
Coronavirus response—active support for hospitalized COVID-19 patients (NCT04343001)	Withdrawn	University College Hospital Ibadan, Oyo, Nigeria, and Shifa Tameer-e-Millat University, Rawalpindi, Pakistan	This project aims to evaluate the effect of aspirin (150 mg once daily), losartan (100 mg once daily), and simvastatin (80 mg once daily) in patients with COVID-19 infection.	No results posted

*Data was acquired as of April 16, 2022. aHR, adjusted hazard ratio (HR); aOR, adjusted odds ratio (OR); BMI, body mass index; CI, confidence interval; ICU, intense care unit; IMV, invasive mechanical ventilation; I^2^, I-squared statistics indicating between-study heterogeneity; RR, risk ratio*.

Different SARS-CoV-2 variants cause resurges of infections (57–60). Vaccines and antiviral therapies are powerful tools against COVID-19 (61, 62), yet data regarding the responses of obese individuals or statin users to these agents remain limited. It has been hypothesized that vaccines would offer reduced protection in obese individuals, based on evidence of immune cell dysregulation and alterations in inflammatory signaling pathways (4, 63). Given the immunomodulatory effects of statins, concerns have also been raised regarding possible interferences with COVID-19 vaccines. Moreover, cautions should be take that drug interactions between statins and some agents used in COVID-19 treatment, may lead to adverse effects (64–66).

Here, we provide a comprehensive update of the values, possible mechanisms, and noteworthy cautions regarding statin use in COVID-19. This review was conducted by consulting resources from peer-reviewed articles and/or official websites like WHO. Common search terms included “COVID-19 OR SARS-CoV-2” AND “statin OR lipid lowering”, etc.

## Values and Mechanisms of Statins in COVID-19

As effective drugs for reducing cholesterol, statins can prevent cardiovascular events which are key risk factors for COVID-19 infection and poor prognosis (9, 67–69). Cholesterol reduction allows statins to affect cell membrane structure and function, particularly lipid rafts that play important roles in viral entry and cellular processes like signal transduction (70–72). Moreover, by reducing intermediate products of cholesterol biosynthesis, statins downregulate protein isoprenylation to regulate numerous signaling pathways including immune responses (73). Hyperactivation of immune responses, elevated systematic inflammation, increased oxidative stress, and thrombosis events have been observed in severe COVID-19, especially among those with obesity or cardiovascular diseases, while statins have shown suppressive effects against these processes (Figure 1).

### Antiviral Effects

**Figure 2:**
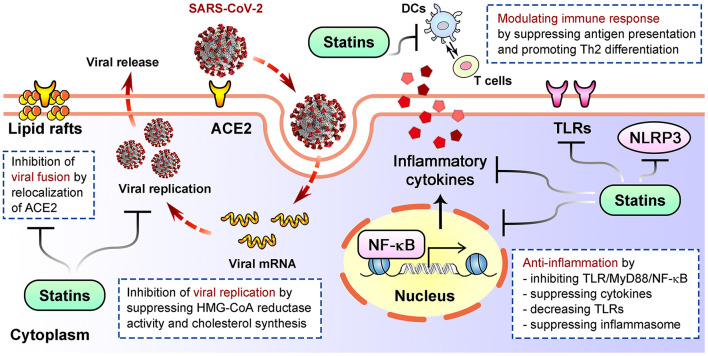
Well established mechanisms of how statins inhibit viral life cycle, alleviate inflammation and modulate immune response after infection. DCs, dendritic cells; TLRs, Toll-like receptors.

SARS-CoV-2 infection initiates from cell entry by attaching to its receptor ACE2 (70, 71). The cholesterol-rich membrane lipid rafts is crucial for this process. By reducing cholesterol, disrupting lipid raft composition, altering membrane receptor assembly and localization, statins interfere with virus fusion and entry in HIV models (51, 74). Potential mechanism is that statins-mediated blockade of HMG-CoA reductase leads to inhibition of Rho guanosine triphosphatase, a key contributor to clustering of lipid raft-associated receptors (74, 75). Statins may increase ACE2 levels under disease situations with unknown clinical relevance (76), and simvastatin significantly affected SARS-CoV-2 cell entry through displacing ACE2 on lipid rafts (77) (Figure 2). Simvastatin can also reduce SARS-CoV-2 replication (77). Viral infection increases HMG-CoA reductase activity and cholesterol synthesis to assist viral replication, which explains the negative impact of statins on viral replication (78, 79) (Figure 2). Viral assembly and release following replication can be suppressed by statins through inhibiting mevalonate synthesis and intracellular cholesterol levels (80, 81). Whether statins similarly affect the assembly and release of SARS-CoV-2 remain unknown.

Although multiple statins have showed antiviral effects against different viruses, a study suggested higher efficacy for lipophilic statins against Zika viral replication, possibly because they can enter cells *via* passive transport to reach higher intracellular concentrations (82). Moreover, comparison between the survival curves of patients with a pre-existing chronic cardiovascular disease indicated a significant reduction in mortality in lipophilic statin users vs. hydrophilic group and non-users group (35). Therefore, it will be critical to understand whether and how the type, dose and duration of statin therapy affect antiviral effects and subsequently the outcome of SARS-CoV-2 infection.

### Anti-inflammatory and Immunomodulatory Effects

Exacerbated inflammation is a pathological hallmark of COVID-19 (83). During severe COVID-19, a generalized inflammatory state is caused by cytokine storm due to hyperactivation of host immune system, leading to lesions in multiple organs and even death (84). Upon SARS-CoV-2 invasion, antigen-presenting cells recognize the pathogen *via* Toll-like receptors (TLRs) and activates two main downstream pathways, MyD88- and TRIF-dependent pathways, both leading to NF-κB activation (85–88). NF-κB initiates the first stage of inflammasome activation and induces the production of pro-inflammatory factors, including interleukin-6 (IL-6), a key cytokine associated with COVID-19 severity and mortality (89–91). Activation of NLRP3 inflammasome involves in response to infection of RNA viruses including SARS-CoV-2 (92–95). Patients with a reduced immune fitness and pre-existing systemic inflammatory state, such as obesity or cardiovascular diseases, are prone to demonstrate dysregulated NLRP3 inflammasome activity and pro-inflammatory cytokines expression, resulting in severe COVID-19 (92, 96, 97).

Statins are known for anti-inflammatory and immunomodulatory effects (Figure 2). Statins can decrease TLRs expression, suppress MYD88-NF-κB pathway and the levels of pro-inflammatory cytokines like IL-6, IL-8, TNF-α and MCP-1, thereby altering inflammatory pathway to reduce cell damage (21, 98–100). Statins directly regulate NLRP3 inflammasome (101), or suppress TLR4-MyD88-NF-κB pathway to inhibit its activation (102), thus downregulate cytokines including IL-18 and IL-1β (103, 104). Immunomodulatory actions also underlie statins' beneficial effects in pathologic status. For examples, statins block mevalonate generation required for T cell proliferation (105), repress MHC-II molecules that are critical for presenting antigen to T cells (23, 106), and suppress maturation of dendritic cells (107), therefore may alleviate hyperactivation of immune response. Rosuvastatin promotes the differentiation of peripheral blood monocytes into anti-inflammatory M2 macrophages (49, 108); atorvastatin suppresses proliferation of naïve Th0 cells and secretion of Th1 pro-inflammatory cytokines, while enhances secretion of Th2 anti-inflammatory cytokines (109).

Clinical studies indicate that statins decrease serum CRP levels (110), an inflammatory biomarker and risk factor for adverse COVID-19 outcomes (111). Importantly, in-hospital statin use is significantly associated with ameliorated inflammatory responses, as reflected by lower levels of circulating CRP, IL-6 and neutrophil counts in statin users (29). Correspondingly, simvastatin downregulated SARS-CoV-2-infection-triggered inflammation in human neutrophils, peripheral blood monocytes, and lung epithelial Calu-3 cells, showing its anti-inflammatory effect both at the site of viral infection and systemically (77). Statin-mediated CRP reduction can be achieved by lowering LDL-C, suppressing Rac-1 activation and increasing apolipoprotein A-I, all of which alleviate inflammation and subsequent CRP generation (112). Notably, for PCSK9 inhibitors, another class of lipid-lowering drugs that significantly decreases LDL-C but not inflammatory markers like CRP (113, 114), evidence is lacked so far regarding their possible benefits on COVID-19 outcomes, while deeper investigation is needed. Since COVID-19 patients with obesity are prone to immune cell dysregulation and elevated inflammations, further studies are warranted to explore whether and how statins may protect them from COVID-19.

### Anti-thrombosis Effects

Thrombosis are among the most frequent complications in COVID-19 patients, especially in critically ill cases (115–117), and elevated D-dimer levels show prognostic significance for poor outcomes (7, 116, 118). Therefore, prevention/alleviation of thrombosis is a key to COVID-19 treatment, especially for obese patients who are prone to ICU admission and thromboembolic events.

Statins can reduce the occurrence of deep vein thrombosis and pulmonary embolism (52, 119–122), common thrombotic events in COVID-19 cases (123, 124). The anti-thrombosis impact of statins not only relates to its cholesterol-lowering effects and to plaque stabilization (125), but also involves lipid-lowering independent inhibitory effect on platelets activation and coagulation cascade, major pathways for thrombosis formation (18, 52, 126). Statins exert antiplatelet effect *via* downregulating prothrombotic factors including platelet thromboxane A2, NOX2 (the catalytic subunit of NADPH oxidase), oxidized low-density lipoprotein (oxLDL) and its receptor CD36 (127–129), and *via* promoting endothelial nitric oxide synthase (eNOS) which improves production of platelet nitric oxide (NO), a potent inhibitor of platelet activation and aggregation (130, 131).

Statins also interfere with clotting system and coagulation cascade. Statins downregulate the expression and activity of tissue factor which initiates the extrinsic pathway of coagulation (132–136), and reduce the serum level of plasminogen activator inhibitor (137); meanwhile, statins upregulate KLF2 that has anticoagulant and atheroprotective effects (138, 139), promote thrombomodulin expression and fibrinolytic activity (140–143). Since a mutual relationship exists between immune activation and thrombus formation (144), the anti-inflammatory actions of statins may also contribute to thrombosis suppression (18).

The anti-thrombosis effect of statins has been widely investigated in patients at risk for cardiovascular disease or those with established atherosclerosis, and varies from different statins (145, 146), but their impacts on COVID-19-related thrombosis remain largely unknown. A clinical trial in ICU admitted COVID-19 patients observed lower rate of thrombosis event in atorvastatin group, although without significant association, suggesting possible anti-thrombosis role of atorvastatin in COVID-19 cases; assessment of outcomes after long-time follow-up is ongoing (46).

### Anti-oxidative Effects

Excessive reactive oxygen species (ROS) is associated with high neutrophil to lymphocyte ratio in critically ill COVID-19 (147). In monocytes and macrophages, SARS-CoV-2 infection triggers mitochondrial ROS production, induces HIF1α stabilization and consequently promotes glycolysis which facilitates viral replication (148). Overwhelming oxidative stress also causes local or systemic damages, induces thrombosis, contributing to COVID-19 severity (149).

Statins exerts anti-oxidative effect by attenuating NF-κB activation, reducing circulating oxLDL and their uptake by macrophages, inhibiting oxidant enzymes such as NADPH oxidase and myeloperoxidase, and upregulating the activity of antioxidant enzymes like catalase and paraoxonase (53, 150). Additionally, statins downregulate NOX2-derived oxidative stress, ultimately exerting antiplatelet effects (128, 151–153). Despite these anti-oxidative effects which possibly benefits COVID-19 treatment, statins may induce ROS production, mediate redox imbalance and consequent cellular oxidative damage, especially under excessive or long-term statin use (154).

## Clinical Trials Regarding Statin Use and COVID-19

Currently, among clinical trials regarding statin use and COVID-19, two have published results, while the others remain uncompleted or have not posted results (Table 1). In INSPIRATION/INSPIRATION-S study (NCT04486508) conducted in Iran ICU admitted COVID-19 patients, atorvastatin (20 mg/day) was not associated with a significant reduction in the composite of thrombosis, ECMO treatment, or all-cause mortality; however, atorvastatin treatment was safe, and may have clinical importance with lower overall event rates (46). Another study (NCT04359095) was conducted in Colombia (47), emtricitabine with tenofovir disoproxil (200/300 mg/day for 10 days) plus colchicine and rosuvastatin (0.5 mg and 40 mg/day for 14 days) combination reduced the risk of 28-day all-cause mortality by 22%, and lowered the need for IMV (47). These findings indicated safety and potential benefits of statins for COVID-19 treatment, yet the therapeutic effect varies from cohort and medications. More randomized controlled trials are therefore warranted to assess the effect of statin administration alone or in combination with regards of medication dose and duration, and to evaluate the influence of chronic statin use on COVID-19-related in-hospital events or long-term complications.

## Cautions About Statins Use in COVID-19

**Figure 3:**
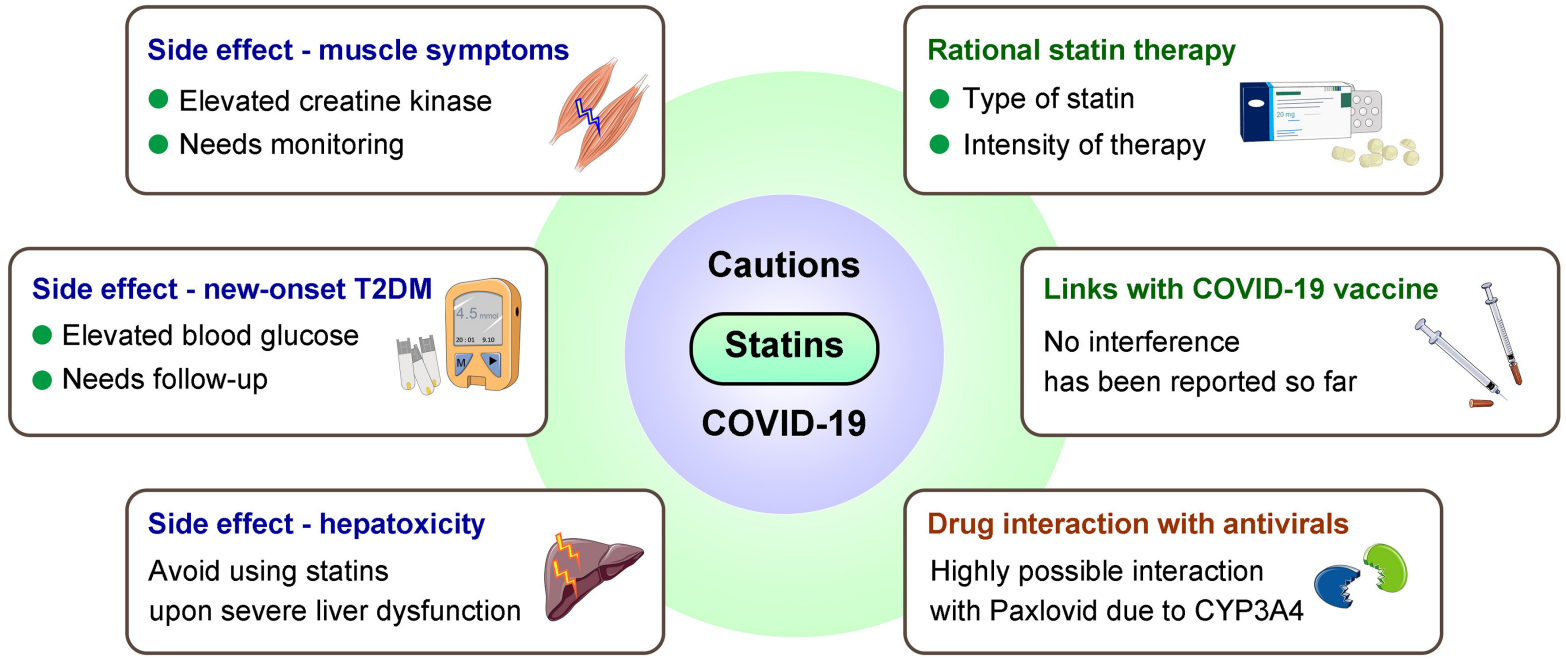
Major cautions for statin use in COVID-19 patients. Considerations are needed from aspects including side effects, interferences with COVID-19 vaccines/antivirals, and proper statin therapy. T2DM, type 2 diabetes mellitus.

Although statins are generally well-tolerated, for COVID-19 patients especially those with obese or chronic statin use, cautions are required for potential risks of statins-associated muscle symptoms, liver injury, new-onset diabetes, renal injury, and neurological and neurocognitive disorders, which may also result from severe COVID-19 (68, 84, 126, 155, 156) (Figure 3).

Statin-associated muscular symptoms (SAMS) are principal cause of poor patient compliance that contribute to adverse outcomes (157, 158). SAMS include fatigue, weakness and pain, possibly accompanied by elevated serum CK levels and activity (54), while similar symptoms also present at early onset of COVID-19 (159). Therefore, for COVID-19 patients who use statins, careful monitoring muscle symptoms and CK levels are necessary; when muscle symptoms occur, assessment and approaches for statin intolerance may be considered (68, 160).

Statins-associated hepatotoxicity may add to COVID-19 related liver injury that potentially caused by psychological stress, systemic inflammation, etc., especially among obese individuals at higher risk of liver dysfunction (161–163). It has been suggested to avoid statin use in the case of severe liver damages, liver failure, and decompensated cirrhosis (24).

COVID-19 may induce or accelerate type 2 diabetes mellitus (T2DM) development as one of its acute and suspected long-term complications (19, 56), while statin may increase incidence of new-onset T2DM, which appears to be more common in obese patients (55, 164, 165). Despite the risk of T2DM, the cardiovascular benefits of statins should not be masked (166). Therefore, statin therapy can be continued in such patients with glucose monitoring, to achieve better glycemic control and avoid developing metabolic disorders after SARS-CoV-2 infection.

Clinicians should also be cautious when treating statin users with COVID-19 who show renal or neurological symptoms and relevant laboratory abnormalities. Renal dysfunction can be caused by SARS-CoV-2 infection and is associated with COVID-19 poor prognosis (167–169); whether statin-associated renal toxicity (170, 171) exacerbates COVID-19-related renal dysfunction remains unclear. There are also concerns regarding whether use of statin (especially lipophilic ones that can cross the blood-brain barrier) may worsen clinical manifestations of nervous system in COVID-19 patients, given their side effects of causing neurological disorders (171, 172).

Caution is also necessary for drug interaction during COVID-19 management. Most statins are predominantly metabolized by CYP450 enzymes, mainly through CYP3A4 (64). Therefore, concomitant administration of CYP3A4 inhibitors, such as some macrolides (clarithromycin/telithromycin/erythromycin) and antiretroviral drugs (lopinavir/ritonavir), with statins can increase the risk of adverse events (68, 173). For severe COVID-19 patients treated with IL-6 receptor blocker tocilizumab, rosuvastatin is recommended (68). Additionally, decreased LDL-C was observed in some severe COVID-19 cases (174, 175). Such finding may due to a more intensive lipid-lowering treatment in patients with high cardiovascular risk who are more vulnerable to COVID-19 (176), and deeper analysis is warranted to better prescribe appropriate intensity statin therapy.

## Statins and COVID-19 Vaccines

The impact of statins on vaccine efficacy remain controversy. Take influenza vaccine for example, a clinical trial suggested immunosuppressive effect of chronic statin medication may weaken the immune response to vaccine (177), whereas another study indicated that statin did not modify influenza vaccine effectiveness (178). Presently, multiple COVID-19 vaccines have been approved, notably, vaccination participants with BMI ≥ 30 had a smaller infection risk reduction than those with BMI < 30 (179), and central obesity (higher waist circumference) is associated with lower neutralizing antibody titers following vaccination (180). Presently, no evidence suggests statin may affect COVID-19 vaccine effectiveness.

## Statins and COVID-19 Antivirals

SARS-CoV-2 variants may have substantial immune evasiveness that weakens vaccine protection due to spike protein mutations (57–60). Small molecular antivirals have reduced hospitalization rate and mortality in patients with promising safety (181, 182), among which Paxlovid stands out by reducing the risk of COVID-19-related hospital admission or death by 89% (183, 184). Paxlovid consists of a SARS-CoV-2 main protease inhibitor PF-07321332, and an anti-HIV drug ritonavir that boosts the effectiveness of protease inhibitors (184). However, co-administration of statins and Paxlovid may increase statin exposure and the risk of adverse effects including muscle symptoms and liver toxicity, because ritonavir potently inhibits CYP3A4 through which lipophilic statins are predominantly metabolized (64–66). Therefore, when such concomitant use is needed, it is possible to continue rosuvastatin therapy starting a low dose and titrating up (68, 156). More studies are needed to clarify the possible risks regarding co-administration of statins and antivirals, to find a proper regimen, and to explore whether obesity and dyslipidemia may interfere the efficacy of antivirals.

## Conclusion and Future Perspectives

Current data suggest that statins are safe for COVID-19 patients and may exert therapeutical benefits. Generally, there is no necessity to discontinue statin use, and no evidence suggesting interference between statins and COVID-19 vaccines. However, cautions should be taken to achieve proper medication for statin users with COVID-19, considering possible side effects and drug interaction (Figure 3). Two major cautions are: *Proper type of statin*. Compared to hydrophilic statins, lipophilic statins enter cells *via* passive transport to reach higher intracellular concentrations, and have a larger distribution volume, thus may be more protective in respect of anti-viral ability. *Intensity of statin therapy*. Hypolipidemia is harmful. Decreased LDL-C is observed in some COVID-19 patients and associated with COVID-19 severity (174, 175), yet such findings may due to a more intensive lipid-lowering treatment in patients with high cardiovascular risk who are more vulnerable to COVID-19 (176). Low-, moderate-, high-intensity statin therapy should be applied according to specific LDL-C lowering needs. Importantly, careful monitoring of LDL-C, CK, blood glucose and liver function is recommended in context of COVID-19.

Currently known impacts of statins in COVID-19 are mostly based on observational studies and may vary due to heterogenicity in different trials/cohorts. To address the effect of statins in COVID-19 patients, especially in those with obesity or dyslipidemia-related diseases, randomized controlled trials with proper patient stratification are warranted. Moreover, experimental evidence of how different statins act in COVID-19 models are rare, highlighting the importance of related studies.

## Author Contributions

CL, WY, YC, and KH: conceptualization. CL, WY, YC, and JS: writing—original draft preparation. CL, YC, SW, AP, and KH: writing—review and editing. All authors have read and agreed to the final version of the manuscript.

## Funding

This work was supported by the Natural Science Foundation of China (31971066), the China Postdoctoral Science Foundation (2021M700050), the Natural Science Foundation of Hubei Province (2021CFA004 and 2021CFB250), and the Postdoctoral Innovation Research Program of Hubei Province.

## Conflict of Interest

The authors declare that the research was conducted in the absence of any commercial or financial relationships that could be construed as a potential conflict of interest.

## Publisher's Note

All claims expressed in this article are solely those of the authors and do not necessarily represent those of their affiliated organizations, or those of the publisher, the editors and the reviewers. Any product that may be evaluated in this article, or claim that may be made by its manufacturer, is not guaranteed or endorsed by the publisher.

## References

[1] Cai QX Chen FJ Wang T Luo F Liu XH Wu QK. Obesity and Covid-19 severity in a designated hospital in Shenzhen, China. Diabetes Care. (2020) 43:1392–8. 10.2337/dc20-0576 32409502

[2] Docherty AB Harrison EM Green CA Hardwick HE Pius R Norman L. Features of 20133 Uk patients in hospital with Covid-19 using the isaric who clinical characterisation protocol: prospective observational cohort study. BMJ. (2020) 369:m1985. 10.1136/bmj.m1985 32444460 PMC7243036

[3] Lighter J Phillips M Hochman S Sterling S Johnson D Francois F. Obesity in patients younger than 60 years is a risk factor for Covid-19 hospital admission. Clin Infect Dis. (2020) 71:896–7. 10.1093/cid/ciaa415 32271368 PMC7184372

[4] Popkin BM Du SF Green WD Beck MA Algaith T Herbst CH. Individuals with obesity and Covid-19: a global perspective on the epidemiology and biological relationships. Obes Rev. (2020) 21:e13128. 10.1111/obr.13128 32845580 PMC7461480

[5] Simonnet A Chetboun M Poissy J Raverdy V Noulette J Duhamel A. High prevalence of obesity in severe acute respiratory syndrome coronavirus-2 (SARS-CoV-2) requiring invasive mechanical ventilation. Obesity. (2020) 28:1195–9. 10.1002/oby.22831 32271993 PMC7262326

[6] Palaiodimos L Kokkinidis DG Li WJ Karamanis D Ognibene J Arora S. Severe obesity, increasing age and male sex are independently associated with worse in -hospital outcomes, and higher in -hospital mortality, in a Cohort of patients with Covid-19 in the Bronx, New York. Metabolism. (2020) 108:154262. 10.1016/j.metabol.2020.154262 32422233 PMC7228874

[7] Zhou F Yu T Du R Fan G Liu Y Liu Z. Clinical course and risk factors for mortality of adult inpatients with Covid-19 in Wuhan, China: a retrospective cohort study. Lancet. (2020) 395:1054–62. 10.1016/S0140-6736(20)30566-3 32171076 PMC7270627

[8] Wu C Chen X Cai Y Xia J Zhou X Xu S. Risk factors associated with acute respiratory distress syndrome and death in patients with coronavirus disease 2019. Pneumonia in Wuhan, China. JAMA Intern Med. (2020) 180:934–43. 10.1001/jamainternmed.2020.0994 32167524 PMC7070509

[9] Vuorio A Kaste M Kovanen PT. Familial hypercholesterolemia and statins in the Covid-19 era: mitigating the risk of ischemic stroke. eNeurologicalSci. (2021) 23:100344. 10.1016/j.ensci.2021.100344 33937536 PMC8078044

[10] Oxley TJ Mocco J Majidi S Kellner CP Shoirah H Singh IP. Large-vessel stroke as a presenting feature of covid-19 in the young. N Engl J Med. (2020) 382:e60. 10.1056/NEJMc2009787 32343504 PMC7207073

[11] Zhang W Yang D Yuan Y Liu C Chen H Zhang Y. Muscular G9a regulates muscle-liver-fat axis by musclin under overnutrition in female mice. Diabetes. (2020) 69:2642–54. 10.2337/db20-0437 32994276

[12] Chen H Liu C Cheng C Zheng L Huang K. Effects of apelin peptides on diabetic complications. Curr Protein Pept Sci. (2018) 19:179–89. 10.2174/1389203718666170918154728 28925900

[13] Grasselli G Zangrillo A Zanella A Antonelli M Cabrini L Castelli A. Baseline characteristics and outcomes of 1591. Patients infected with SARS-CoV-2 admitted to icus of the Lombardy Region, Italy. JAMA. (2020) 323:1574–81. 10.1001/jama.2020.5394 32250385 PMC7136855

[14] Petrilli CM Jones SA Yang J Rajagopalan H O'Donnell L Chernyak Y. Factors associated with hospital admission and critical illness among 5279 people with coronavirus disease 2019 in New York City: prospective cohort study. BMJ. (2020) 369:m1966. 10.1136/bmj.m1966 32444366 PMC7243801

[15] Casas-Rojo JM Anton-Santos JM Millan-Nunez-Cortes J Lumbreras-Bermejo C Ramos-Rincon JM Roy-Vallejo E. Clinical characteristics of patients hospitalized with covid-19 in Spain: results from the semi-Covid-19 registry. Rev Clin Esp. (2020) 220:480–94. 10.1016/j.rceng.2020.07.003 33994573 PMC7368900

[16] Ghoneim S Butt MU Hamid O Shah A Asaad I. The incidence of Covid-19 in patients with metabolic syndrome and non-alcoholic steatohepatitis: a population-based study. Metabol Open. (2020) 8:100057. 10.1016/j.metop.2020.100057 32924000 PMC7480663

[17] Gomez-Mesa JE Galindo-Coral S Montes MC Munoz Martin AJ. Thrombosis and **c**oagulopathy in Covid-19. Curr Probl Cardiol. (2021) 46:100742. 10.1016/j.cpcardiol.2020.100742 33243440 PMC7605852

[18] Gasecka A Borovac JA Guerreiro RA Giustozzi M Parker W Caldeira D. Thrombotic complications in patients with covid-19: pathophysiological mechanisms, diagnosis, and treatment. Cardiovasc Drugs Ther. (2021) 35:215–29. 10.1007/s10557-020-07084-9 33074525 PMC7569200

[19] Hayden MR. An immediate and long-term complication of covid-19 may be type 2 diabetes mellitus: the central role of cell dysfunction, apoptosis and exploration of possible mechanisms. Cells. (2020) 9:2475. 10.3390/cells9112475 33202960 PMC7697826

[20] Blais JE Wei Y Yap KKW Alwafi H Ma TT Brauer R. Trends in lipid-modifying agent use in 83 countries. Atherosclerosis. (2021) 328:44–51. 10.1016/j.atherosclerosis.2021.05.016 34091069

[21] Lefer DJ. Statins as potent antiinflammatory drugs. Circulation. (2002) 106:2041–2. 10.1161/01.CIR.0000033635.42612.88 12379569

[22] Igel M Sudhop T von Bergmann K. Pharmacology of 3-hydroxy-3-methylglutaryl-coenzyme a reductase inhibitors (statins), including rosuvastatin and pitavastatin. J Clin Pharmacol. (2002) 42:835–45. 10.1177/009127002401102731 12162466

[23] Kwak B Mulhaupt F Myit S Mach F. Statins as a newly recognized type of immunomodulator. Nat Med. (2000) 6:1399–402. 10.1038/82219 11100127

[24] Meurer L Cohen SM. Drug-induced liver injury from statins. Clin Liver Dis. (2020) 24:107–19. 10.1016/j.cld.2019.09.007 31753243

[25] Kazi DS Penko JM Bibbins-Domingo K. Statins for primary prevention of cardiovascular disease: review of evidence and recommendations for clinical practice. Med Clin North Am. (2017) 101:689–99. 10.1016/j.mcna.2017.03.001 28577620

[26] Bibbins-Domingo K Grossman DC Curry SJ Davidson KW Epling JW Jr. García FAR. Statin use for the primary prevention of cardiovascular disease in adults: US preventive services task force recommendation statement. JAMA. (2016) 316:1997–2007. 10.1001/jama.2016.15450 27838723

[27] Song SL Hays SB Panton CE Mylona EK Kalligeros M Shehadeh F. Statin use is associated with decreased risk of invasive mechanical ventilation in covid-19 patients: a preliminary study. Pathogens. (2020) 9:759. 10.3390/pathogens9090759 32957539 PMC7559887

[28] Vahedian-Azimi A Mohammadi SM Heidari Beni F Banach M Guest PC Jamialahmadi T. Improved covid-19 ICU admission and mortality outcomes following treatment with statins: a systematic review and meta-analysis. Arch Med Sci. (2021) 17:579–95. 10.5114/aoms/132950 34025827 PMC8130467

[29] Zhang XJ Qin JJ Cheng X Shen L Zhao YC Yuan Y. In-hospital use of statins is associated with a reduced risk of mortality among individuals with covid-19. Cell Metab. (2020) 32:176–87.e4. 10.1016/j.cmet.2020.06.015 32592657 PMC7311917

[30] Lee HY Ahn J Park J Kyung Kang C Won SH Wook Kim D. Beneficial effect of statins in covid-19-related outcomes-brief report: a national population-based cohort study. Arterioscler Thromb Vasc Biol. (2021) 41:e175–e82. 10.1161/ATVBAHA.120.315551 33535790 PMC7901529

[31] Memel ZN Lee JJ Foulkes AS Chung RT Thaweethai T Bloom PP. Association of statins and 28-day mortality rates in patients hospitalized with severe acute respiratory syndrome coronavirus 2 infection. J Infect Dis. (2022) 225:19–29. 10.1093/infdis/jiab539 34665852 PMC8586726

[32] Choi D Chen Q Goonewardena SN Pacheco H Mejia P Smith RL. Efficacy of statin therapy in patients with hospital admission for covid-19. Cardiovasc Drugs Ther. (2021). 10.1007/s10557-021-07263-2. [Epub ahead of print]. 34524566 PMC8440735

[33] Rodriguez-Nava G Trelles-Garcia DP Yanez-Bello MA Chung CW Trelles-Garcia VP Friedman HJ. Atorvastatin associated with decreased hazard for death in covid-19 patients admitted to an icu: a retrospective cohort study. Crit Care. (2020) 24:429. 10.1186/s13054-020-03154-4 32664990 PMC7358561

[34] Daniels LB Sitapati AM Zhang J Zou J Bui QM Ren J. Relation of statin use prior to admission to severity and recovery among covid-19 inpatients. Am J Cardiol. (2020) 136:149–55. 10.1016/j.amjcard.2020.09.012 32946859 PMC7492151

[35] Rossi R Talarico M Coppi F Boriani G. Protective role of statins in covid 19 patients: importance of pharmacokinetic characteristics rather than intensity of action. Intern Emerg Med. (2020) 15:1573–6. 10.1007/s11739-020-02504-y 33011928 PMC7532733

[36] Saeed O Castagna F Agalliu I Xue X Patel SR Rochlani Y. Statin use and in-hospital mortality in patients with diabetes mellitus and covid-19. J Am Heart Assoc. (2020) 9:e018475. 10.1161/JAHA.120.018475 33092446 PMC7955378

[37] De Spiegeleer A Bronselaer A Teo JT Byttebier G De Tré G Belmans L. The effects of arbs, aceis, and statins on clinical outcomes of covid-19 infection among nursing home residents. J Am Med Dir Assoc. (2020) 21:909–14.e2. 10.1016/j.jamda.2020.06.018 32674818 PMC7294267

[38] Lala A Johnson KW Januzzi JL Russak AJ Paranjpe I Richter F. Prevalence and impact of myocardial injury in patients hospitalized with covid-19 infection. J Am Coll Cardiol. (2020) 76:533–46. 10.1101/2020.04.20.20072702 32517963 PMC7279721

[39] Gupta A Madhavan MV Poterucha TJ DeFilippis EM Hennessey JA Redfors B. Association between antecedent statin use and decreased mortality in hospitalized patients with covid-19. Nat Commun. (2021) 12:1325. 10.1038/s41467-021-21553-1 33637713 PMC7910606

[40] Byttebier G Belmans L Alexander M Saxberg BEH De Spiegeleer B De Spiegeleer A. Hospital mortality in covid-19 patients in belgium treated with statins, ace inhibitors and/or arbs. Hum Vaccin Immunother. (2021) 17:2841–50. 10.1080/21645515.2021.1920271 34047686 PMC8171011

[41] Ayeh SK Abbey EJ Khalifa BAA Nudotor RD Osei AD Chidambaram V. Statins use and covid-19 outcomes in hospitalized patients. PLoS ONE. (2021) 16:e0256899. 10.1371/journal.pone.0256899 34506533 PMC8432819

[42] Kollias A Kyriakoulis KG Kyriakoulis IG Nitsotolis T Poulakou G Stergiou GS. Statin use and mortality in covid-19 patients: updated systematic review and meta-analysis. Atherosclerosis. (2021) 330:114–21. 10.1016/j.atherosclerosis.2021.06.911 34243953 PMC8233054

[43] Lee SW Kim SY Moon SY Yoo IK Yoo EG Eom GH. Statin use and covid-19 infectivity and severity in South Korea: two population-based nationwide cohort studies. JMIR Public Health Surveill. (2021) 7:e29379. 10.2196/29379 34623311 PMC8510150

[44] Kow CS Hasan SS. Meta-analysis of effect of statins in patients with covid-19. Am J Cardiol. (2020) 134:153–5. 10.1016/j.amjcard.2020.08.004 32891399 PMC7419280

[45] Tan WYT Young BE Lye DC Chew DEK Dalan R. Statin use is associated with lower disease severity in covid-19 infection. Sci Rep. (2020) 10:17458. 10.1038/s41598-020-74492-0 33060704 PMC7562925

[46] INSPIRATION-S Investigators. Atorvastatin versus placebo in patients with covid-19 in intensive care: randomized controlled trial. BMJ. (2022) 376:e068407. 10.1136/bmj-2021-068407 34996756 PMC11785411

[47] Gaitan-Duarte HG Alvarez-Moreno C Rincon-Rodriguez CJ Yomayusa-Gonzalez N Cortes JA Villar JC. Effectiveness of rosuvastatin plus colchicine, emtricitabine/tenofovir and combinations thereof in hospitalized patients with covid-19: a pragmatic, open-label randomized trial. EClinicalMedicine. (2022) 43:101242. 10.1016/j.eclinm.2021.101242 34957385 PMC8686571

[48] Flumignan RL Civile VT Tinôco JDS Pascoal PI Areias LL Matar CF. Anticoagulants for people hospitalised with covid-19. Cochrane Database Syst Rev. (2022) 3:Cd013739. 10.1002/14651858.CD013739.pub2 35244208 PMC8895460

[49] Kashour T Halwani R Arabi YM Sohail MR O'Horo JC Badley AD. Statins as an adjunctive therapy for covid-19: the biological and clinical plausibility. Immunopharmacol Immunotoxicol. (2021) 43:37–50. 10.1080/08923973.2020.1863984 33406943

[50] Pawlos A Niedzielski M Gorzelak-Pabis P Broncel M Wozniak E. Covid-19: direct and indirect mechanisms of statins. Int J Mol Sci. (2021) 22:177. 10.3390/ijms22084177 33920709 PMC8073792

[51] Gorabi AM Kiaie N Bianconi V Jamialahmadi T Al-Rasadi K Johnston TP. Antiviral effects of statins. Prog Lipid Res. (2020) 79:101054. 10.1016/j.plipres.2020.101054 32777243

[52] Violi F Calvieri C Ferro D Pignatelli P. Statins as antithrombotic drugs. Circulation. (2013) 127:251–7. 10.1161/CIRCULATIONAHA.112.145334 23319813

[53] Davignon J Jacob RF Mason RP. The antioxidant effects of statins. Coron Artery Dis. (2004) 15:251–8. 10.1097/01.mca.0000131573.31966.34 15238821

[54] Bouitbir J Sanvee GM Panajatovic MV Singh F Krähenbühl S. Mechanisms of statin-associated skeletal muscle-associated symptoms. Pharmacol Res. (2020) 154:104201. 10.1016/j.phrs.2019.03.010 30877064

[55] Banach M Rizzo M Toth PP Farnier M Davidson MH Al-Rasadi K. Statin Intolerance - an attempt at a unified definition. Position paper from an international lipid expert panel. Arch Med Sci. (2015) 11:1–23. 10.5114/aoms.2015.49807 25861286 PMC4379380

[56] Boddu SK Aurangabadkar G Kuchay MS. New onset diabetes, type 1 diabetes and covid-19. Diabetes Metab Syndr. (2020) 14:2211–7. 10.1016/j.dsx.2020.11.012 33395782 PMC7669477

[57] Wang Z Schmidt F Weisblum Y Muecksch F Barnes CO Finkin S. mRNA vaccine-elicited antibodies to SARS-CoV-2 and circulating variants. Nature. (2021) 592:616–22. 10.1038/s41586-021-03324-6 33567448 PMC8503938

[58] Collier DA De Marco A Ferreira I Meng B Datir RP Walls AC. Sensitivity of SARS-CoV-2 B117 to mRNA vaccine-elicited antibodies. Nature. (2021) 593:136–41. 10.1038/s41586-021-03412-7 33706364 PMC7616976

[59] Sokal A Broketa M Barba-Spaeth G Meola A Fernández I Fourati S. Analysis of mRNA vaccination-elicited Rbd-specific memory B cells reveals strong but incomplete immune escape of the SARS-CoV-2 omicron variant. Immunity. (2022). 10.1016/j.immuni.2022.04.002. [Epub ahead of print]. 35483354 PMC8986479

[60] Wang Z Muecksch F Cho A Gaebler C Hoffmann H-H Ramos V. Analysis of memory B cells identifies conserved neutralizing epitopes on the N-terminal domain of variant SARS-CoV-2 spike proteins. Immunity. (2022). 10.1016/j.immuni.2022.04.003. [Epub ahead of print]. 35447092 PMC8986478

[61] Kozlov M. Why scientists are racing to develop more covid antivirals. Nature. (2022) 601:496. 10.1038/d41586-022-00112-8 35064230

[62] Wang Z Yang L. In the age of omicron variant: paxlovid raises new hopes of covid-19 recovery. J Med Virol. (2022) 94:1766–7. 10.1002/jmv.27540 34936106

[63] Townsend MJ Kyle TK Stanford FC. Covid-19 vaccination and obesity: optimism and challenges. Obesity. (2021) 29:634–5. 10.1002/oby.23131 33506642 PMC7990687

[64] Sirtori CR. The pharmacology of statins. Pharmacol Res. (2014) 88:3–11. 10.1016/j.phrs.2014.03.002 24657242

[65] Kiortsis DN Filippatos TD Mikhailidis DP Elisaf MS Liberopoulos EN. Statin-associated adverse effects beyond muscle and liver toxicity. Atherosclerosis. (2007) 195:7–16. 10.1016/j.atherosclerosis.2006.10.001 17094994

[66] Lee KCH Sewa DW Phua GC. Potential role of statins in covid-19. Int J Infect Dis. (2020) 96:615–7. 10.1016/j.ijid.2020.05.115 32502659 PMC7265877

[67] Endo A. A historical perspective on the discovery of statins. Proc Jpn Acad Ser B Phys Biol Sci. (2010) 86:484–93. 10.2183/pjab.86.484 20467214 PMC3108295

[68] Banach M Penson PE Fras Z Vrablik M Pella D Reiner Z. Brief recommendations on the management of adult patients with familial hypercholesterolemia during the covid-19 pandemic. Pharmacol Res. (2020) 158:104891. 10.1016/j.phrs.2020.104891 32389859 PMC7204727

[69] Driggin E Madhavan MV Bikdeli B Chuich T Laracy J Biondi-Zoccai G. Cardiovascular considerations for patients, health care workers, and health systems during the covid-19 pandemic. J Am Coll Cardiol. (2020) 75:2352–71. 10.1016/j.jacc.2020.03.031 32201335 PMC7198856

[70] Li Y Xiao Y Chen Y Huang K. Nano-based approaches in the development of antiviral agents and vaccines. Life Sci. (2021) 265:118761. 10.1016/j.lfs.2020.118761 33189824 PMC7658595

[71] Gheblawi M Wang K Viveiros A Nguyen Q Zhong JC Turner AJ. Angiotensin converting enzyme 2: SARS-CoV-2 receptor and regulator of the renin-angiotensin system. Circ Res. (2020) 126:1456–74. 10.1161/CIRCRESAHA.120.317015 32264791 PMC7188049

[72] Wang S Zheng J Ma L Petersen RB Xu L Huang K. Inhibiting protein aggregation with nanomaterials: the underlying mechanisms and impact factors. Biochim Biophys Acta Gen Subj. (2022) 1866:130061. 10.1016/j.bbagen.2021.130061 34822925

[73] Matzinger P. The danger model: a renewed sense of self. Science. (2002) 296:301–5. 10.1126/science.1071059 11951032

[74] del Real G Jiménez-Baranda S Mira E Lacalle RA Lucas P Gómez-Moutón C. Statins inhibit Hiv-1 infection by down-regulating rho activity. J Exp Med. (2004) 200:541–7. 10.1084/jem.20040061 15314078 PMC2211926

[75] Gower TL Graham BS. Antiviral activity of lovastatin against respiratory syncytial virus *in vivo* and *in vitro*. Antimicrob Agents Chemother. (2001) 45:1231–7. 10.1128/AAC.45.4.1231-1237.2001 11257039 PMC90448

[76] South AM Diz DI Chappell MC. Covid-19, Ace2, and the cardiovascular consequences. Am J Physiol Heart Circ Physiol. (2020) 318:H1084–h90. 10.1152/ajpheart.00217.2020 32228252 PMC7191628

[77] Teixeira L Temerozo JR Pereira-Dutra FS Ferreira AC Mattos M Goncalves BS. Simvastatin downregulates the SARS-CoV-2-induced inflammatory response and impairs viral infection through disruption of lipid rafts. Front Immunol. (2022) 13:820131. 10.3389/fimmu.2022.820131 35251001 PMC8895251

[78] Mackenzie JM Khromykh AA Parton RG. Cholesterol manipulation by west nile virus perturbs the cellular immune response. Cell Host Microbe. (2007) 2:229–39. 10.1016/j.chom.2007.09.003 18005741

[79] Rothwell C Lebreton A Young Ng C Lim JY Liu W Vasudevan S. Cholesterol biosynthesis modulation regulates dengue viral replication. Virology. (2009) 389:8–19. 10.1016/j.virol.2009.03.025 19419745

[80] Liao Z Graham DR Hildreth JE. Lipid rafts and HIV pathogenesis: virion-associated cholesterol is required for fusion and infection of susceptible cells. AIDS Res Hum Retroviruses. (2003) 19:675–87. 10.1089/088922203322280900 13678470

[81] Shrivastava-Ranjan P Flint M Bergeron É McElroy AK Chatterjee P Albariño CG. Statins suppress ebola virus infectivity by interfering with glycoprotein processing. mBio. (2018) 9:e00660-18. 10.1128/mBio.00660-18 29717011 PMC5930306

[82] Españo E Nam JH Song EJ Song D Lee CK Kim JK. Lipophilic statins inhibit zika virus production in vero cells. Sci Rep. (2019) 9:11461. 10.1038/s41598-019-47956-1 31391514 PMC6685969

[83] Mehta P McAuley DF Brown M Sanchez E Tattersall RS Manson JJ. Covid-19: consider cytokine storm syndromes and immunosuppression. Lancet. (2020) 395:1033–4. 10.1016/S0140-6736(20)30628-0 32192578 PMC7270045

[84] Vitiello A Ferrara F. Plausible positive effects of statins in covid-19 patient. Cardiovasc Toxicol. (2021) 21:781–9. 10.1007/s12012-021-09674-x 34255300 PMC8275916

[85] Zhang Y Guo X Yan W Chen Y Ke M Cheng C. ANGPTL8 negatively regulates NF-κB activation by facilitating selective autophagic degradation of Ikkγ. Nat Commun. (2017) 8:2164. 10.1038/s41467-017-02355-w 29255244 PMC5735157

[86] Zhang Y Zheng L Huang K. A new way to regulate inflammation: selective autophagic degradation of Ikkγ mediated by Angptl8. Cell Stress. (2018) 2:66–8. 10.15698/cst2018.03.128 31225468 PMC6558929

[87] Fitzgerald KA Kagan JC. Toll-like receptors and the control of immunity. Cell. (2020) 180:1044–66. 10.1016/j.cell.2020.02.041 32164908 PMC9358771

[88] Khan S Shafiei MS Longoria C Schoggins JW Savani RC Zaki H. SARS-CoV-2 spike protein induces inflammation *via* TLR2-dependent activation of the NF-κB pathway. Elife. (2021) 10:e68563. 10.7554/eLife.68563 34866574 PMC8709575

[89] Coomes EA Haghbayan H. Interleukin-6 in covid-19: a systematic review and meta-analysis. Rev Med Virol. (2020) 30:1–9. 10.1002/rmv.2141 32845568 PMC7460877

[90] Hadjadj J Yatim N Barnabei L Corneau A Boussier J Smith N. Impaired type I interferon activity and inflammatory responses in severe covid-19 patients. Science. (2020) 369:718–24. 10.1126/science.abc6027 32661059 PMC7402632

[91] Zhou Q Cheng C Wei Y Yang J Zhou W Song Q. Usp15 potentiates NF-κB activation by differentially stabilizing Tab2 and Tab3. FEBS J. (2020) 287:3165–83. 10.1111/febs.15202 31903660

[92] van den Berg DF Te Velde AA. Severe covid-19: NLRP3 inflammasome dysregulated. Front Immunol. (2020) 11:1580. 10.3389/fimmu.2020.01580 32670297 PMC7332883

[93] Kelley N Jeltema D Duan Y He Y. The NLRP3 inflammasome: an overview of mechanisms of activation and regulation. Int J Mol Sci. (2019) 20:328. 10.3390/ijms20133328 31284572 PMC6651423

[94] Zhao C Zhao W. NLRP3 Inflammasome-a key player in antiviral responses. Front in Immunol. (2020) 11:211. 10.3389/fimmu.2020.00211 32133002 PMC7040071

[95] Yang Y Wang H Kouadir M Song H Shi F. Recent advances in the mechanisms of NLRP3 inflammasome activation and its inhibitors. Cell Death Dis. (2019) 10:128. 10.1038/s41419-019-1413-8 30755589 PMC6372664

[96] López-Reyes A Martinez-Armenta C Espinosa-Velázquez R Vázquez-Cárdenas P Cruz-Ramos M Palacios-Gonzalez B. NLRP3 inflammasome: the stormy link between obesity and covid-19. Front Immunol. (2020) 11:570251. 10.3389/fimmu.2020.570251 33193349 PMC7662564

[97] Yang J Song QY Niu SX Chen HJ Petersen RB Zhang Y. Emerging roles of angiopoietin-like proteins in inflammation: mechanisms and potential as pharmacological targets. J Cell Physiol. (2022) 237:98–117. 10.1002/jcp.30534 34289108

[98] Zelvyte I Dominaitiene R Crisby M Janciauskiene S. Modulation of inflammatory mediators and ppargamma and NFkappaB expression by pravastatin in response to lipoproteins in human monocytes *in vitro*. Pharmacol Res. (2002) 45:147–54. 10.1006/phrs.2001.0922 11846628

[99] Moutzouri E Tellis CC Rousouli K Liberopoulos EN Milionis HJ Elisaf MS. Effect of simvastatin or its combination with ezetimibe on toll-like receptor expression and lipopolysaccharide - induced cytokine production in monocytes of hypercholesterolemic patients. Atherosclerosis. (2012) 225:381–7. 10.1016/j.atherosclerosis.2012.08.037 23062767

[100] Koushki K Shahbaz SK Mashayekhi K Sadeghi M Zayeri ZD Taba MY. Anti-inflammatory action of statins in cardiovascular disease: the role of inflammasome and toll-like receptor pathways. Clin Rev Allergy Immunol. (2021) 60:175–99. 10.1007/s12016-020-08791-9 32378144 PMC7985098

[101] Parsamanesh N Moossavi M Bahrami A Fereidouni M Barreto G Sahebkar A. NLRP3 inflammasome as a treatment target in atherosclerosis: a focus on statin therapy. Int Immunopharmacol. (2019) 73:146–55. 10.1016/j.intimp.2019.05.006 31100709

[102] Kong F Ye B Lin L Cai X Huang W Huang Z. Atorvastatin suppresses NLRP3 inflammasome activation *via* TLR4/MyD88/NF-κB signaling in Pma-stimulated Thp-1 monocytes. Biomed Pharmacother. (2016) 82:167–72. 10.1016/j.biopha.2016.04.043 27470352

[103] Satoh M Tabuchi T Itoh T Nakamura M. NLRP3 inflammasome activation in coronary artery disease: results from prospective and randomized study of treatment with atorvastatin or rosuvastatin. Clin Sci. (2014) 126:233–41. 10.1042/CS20130043 23944632

[104] Krishnan SM Sobey CG Latz E Mansell A Drummond GR. Il-1β and Il-18: inflammatory markers or mediators of hypertension? Br J Pharmacol. (2014) 171:5589–602. 10.1111/bph.12876 25117218 PMC4290704

[105] Chakrabarti R Engleman EG. Interrelationships between mevalonate metabolism and the mitogenic signaling pathway in T lymphocyte proliferation. J Bio Chem. (1991) 266:12216–22. 10.1016/S0021-9258(18)98884-8 1712015

[106] Axelrod ML Cook RS Johnson DB Balko JM. Biological consequences of Mhc-Ii expression by tumor cells in cancer. Clin Cancer Res. (2019) 25:2392–402. 10.1158/1078-0432.CCR-18-3200 30463850 PMC6467754

[107] Yilmaz A Reiss C Tantawi O Weng A Stumpf C Raaz D. Hmg-Coa reductase inhibitors suppress maturation of human dendritic cells: new implications for atherosclerosis. Atherosclerosis. (2004) 172:85–93. 10.1016/j.atherosclerosis.2003.10.002 14709361

[108] Zhang T Shao B Liu GA. Rosuvastatin promotes the differentiation of peripheral blood monocytes into M2 macrophages in patients with atherosclerosis by activating PPAR-γ. Eur Rev Med Pharmacol Sci. (2017) 21:4464–71. 29077145

[109] Youssef S Stuve O Patarroyo JC Ruiz PJ Radosevich JL Hur EM. The Hmg-coa reductase inhibitor, atorvastatin, promotes a Th2 bias and reverses paralysis in central nervous system autoimmune disease. Nature. (2002) 420:78–84. 10.1038/nature01158 12422218

[110] Albert MA Danielson E Rifai N Ridker PM Investigators P. Effect of statin therapy on C-reactive protein levels: the pravastatin inflammation/CRP evaluation (Prince): a randomized trial and cohort study. JAMA. (2001) 286:64–70. 10.1001/jama.286.1.64 11434828

[111] Smilowitz NR Kunichoff D Garshick M Shah B Pillinger M Hochman JS. C-reactive protein and clinical outcomes in patients with covid-19. Eur Heart J. (2021) 42:2270–9. 10.1093/eurheartj/ehaa1103 33448289 PMC7928982

[112] Arévalo-Lorido JC. Clinical relevance for lowering C-reactive protein with statins. Ann Med. (2016) 48:516–24. 10.1080/07853890.2016.1197413 27355392

[113] Ruscica M Ferri N Macchi C Corsini A Sirtori CR. Lipid lowering drugs and inflammatory changes: an impact on cardiovascular outcomes? Ann Med. (2018) 50:461–84. 10.1080/07853890.2018.1498118 29976096

[114] Ruscica M Tokgözoglu L Corsini A Sirtori CR. Pcsk9 inhibition and inflammation: a narrative review. Atherosclerosis. (2019) 288:146–55. 10.1016/j.atherosclerosis.2019.07.015 31404822

[115] Ali MAM Spinler SA. Covid-19 and thrombosis: from bench to bedside. Trends Cardiovasc Med. (2021) 31:143–60. 10.1016/j.tcm.2020.12.004 33338635 PMC7836332

[116] Al-Samkari H Karp Leaf RS Dzik WH Carlson JCT Fogerty AE Waheed A. Covid-19 and coagulation: bleeding and thrombotic manifestations of SARS-CoV-2 infection. Blood. (2020) 136:489–500. 10.1182/blood.2020006520 32492712 PMC7378457

[117] Ferrari F Martins VM Teixeira M Santos RD Stein R. Covid-19 and thromboinflammation: is there a role for statins? Clinics. (2021) 76:e2518. 10.6061/clinics/2021/e2518 33787678 PMC7955154

[118] Levi M Thachil J Iba T Levy JH. Coagulation abnormalities and thrombosis in patients with covid-19. Lancet Haematol. (2020) 7:e438–e40. 10.1016/S2352-3026(20)30145-9 32407672 PMC7213964

[119] Glynn RJ Danielson E Fonseca FA Genest J Gotto AM Jr. Kastelein JJ. A randomized trial of rosuvastatin in the prevention of venous thromboembolism. N Engl J Med. (2009) 360:1851–61. 10.1056/NEJMoa0900241 19329822 PMC2710995

[120] Doggen CJ Lemaitre RN Smith NL Heckbert SR Psaty BM, HMG. CoA reductase inhibitors and the risk of venous thrombosis among postmenopausal women. J Thromb Haemost. (2004) 2:700–1. 10.1111/j.1538-7836.2004.00696.x 15099273

[121] Khemasuwan D Divietro ML Tangdhanakanond K Pomerantz SC Eiger G. Statins decrease the occurrence of venous thromboembolism in patients with cancer. Am J Med. (2010) 123:60–5. 10.1016/j.amjmed.2009.05.025 20102993

[122] Rodriguez AL Wojcik BM Wrobleski SK Myers DD Jr. Wakefield TW Diaz JA. Statins, inflammation and deep vein thrombosis: a systematic review. J Thromb Thrombolysis. (2012) 33:371–82. 10.1007/s11239-012-0687-9 22278047 PMC3338886

[123] Mackman N. Triggers, targets and treatments for thrombosis. Nature. (2008) 451:914–8. 10.1038/nature06797 18288180 PMC2848509

[124] Hanff TC Mohareb AM Giri J Cohen JB Chirinos JA. Thrombosis in covid-19. Am J Hematol. (2020) 95:1578–89. 10.1002/ajh.25982 32857878 PMC7674272

[125] Pucci A Sheiban I Formato L Celeste A Brscic E Moretti C. *In vivo* coronary plaque histology in patients with stable and acute coronary syndromes: relationships with hyperlipidemic status and statin treatment. Atherosclerosis. (2007) 194:189–95. 10.1016/j.atherosclerosis.2006.07.026 16970947

[126] Subir R Jagat JM Kalyan KG. Pros and cons for use of statins in people with coronavirus disease-19 (Covid-19). Diabetes Metab Syndr. (2020) 14:1225–9. 10.1016/j.dsx.2020.07.011 32683320 PMC7352102

[127] Notarbartolo A Davi G Averna M Barbagallo CM Ganci A Giammarresi C. Inhibition of thromboxane biosynthesis and platelet function by simvastatin in type Iia hypercholesterolemia. Arterioscler Thromb Vasc Biol. (1995) 15:247–51. 10.1161/01.ATV.15.2.247 7749833

[128] Puccetti L Santilli F Pasqui AL Lattanzio S Liani R Ciani F. Effects of atorvastatin and rosuvastatin on thromboxane-dependent platelet activation and oxidative stress in hypercholesterolemia. Atherosclerosis. (2011) 214:122–8. 10.1016/j.atherosclerosis.2010.10.006 21056418

[129] Owens AP II Mackman N. The antithrombotic effects of statins. Annu Rev Med. (2014) 65:433–45. 10.1146/annurev-med-051812-145304 24422578

[130] Laufs U Gertz K Huang P Nickenig G Bohm M Dirnagl U. Atorvastatin upregulates type iii nitric oxide synthase in thrombocytes, decreases platelet activation, and protects from cerebral ischemia in normocholesterolemic mice. Stroke. (2000) 31:2442–9. 10.1161/01.STR.31.10.2442 11022078

[131] Haramaki N Ikeda H Takenaka K Katoh A Sugano R Yamagishi S. Fluvastatin alters platelet aggregability in patients with hypercholesterolemia: possible improvement of intraplatelet redox imbalance *via* HMG-CoA reductase. Arterioscler Thromb Vasc Biol. (2007) 27:1471–7. 10.1161/ATVBAHA.106.128793 17379842

[132] Ferro D Basili S Alessandri C Mantovani B Cordova C Violi F. Simvastatin reduces monocyte-tissue-factor expression type Iia hypercholesterolaemia. Lancet. (1997) 350:1222. 10.1016/S0140-6736(05)63452-6 9652566

[133] Colli S Eligini S Lalli M Camera M Paoletti R Tremoli E. Vastatins inhibit tissue factor in cultured human macrophages. A novel mechanism of protection against atherothrombosis. Arterioscler Thromb Vasc Biol. (1997) 17:265–72. 10.1161/01.ATV.17.2.265 9081680

[134] Meisel SR Xu XP Edgington TS Cercek B Ong J Kaul S. Dose-dependent modulation of tissue factor protein and procoagulant activity in human monocyte-derived macrophages by oxidized low density lipoprotein. J Atheroscler Thromb. (2011) 18:596–603. 10.5551/jat.7179 21467727

[135] Markle RA Han J Summers BD Yokoyama T Hajjar KA Hajjar DP. Pitavastatin alters the expression of thrombotic and fibrinolytic proteins in human vascular cells. J Cell Biochem. (2003) 90:23–32. 10.1002/jcb.10602 12938153

[136] Mackman N Tilley RE Key NS. Role of the extrinsic pathway of blood coagulation in hemostasis and thrombosis. Arterioscler Thromb Vasc Biol. (2007) 27:1687–93. 10.1161/ATVBAHA.107.141911 17556654

[137] Sahebkar A Catena C Ray KK Vallejo-Vaz AJ Reiner Ž Sechi LA. Impact of statin therapy on plasma levels of plasminogen activator inhibitor-1. A systematic review and meta-analysis of randomised controlled trials. Thromb Haemost. (2016) 116:162–71. 10.1160/TH15-10-0770 27009446

[138] Lin Z Kumar A SenBanerjee S Staniszewski K Parmar K Vaughan DE. Kruppel-like factor 2 (Klf2) regulates endothelial thrombotic function. Circ Res. (2005) 96:e48–57. 10.1161/01.RES.0000159707.05637.a1 15718498

[139] Sen-Banerjee S Mir S Lin Z Hamik A Atkins GB Das H. Kruppel-like factor 2 as a novel mediator of statin effects in endothelial cells. Circulation. (2005) 112:720–6. 10.1161/CIRCULATIONAHA.104.525774 16043642

[140] Lin SJ Chen YH Lin FY Hsieh LY Wang SH Lin CY. Pravastatin induces thrombomodulin expression in tnfalpha-treated human aortic endothelial cells by inhibiting Rac1 and Cdc42 translocation and activity. J Cell Biochem. (2007) 101:642–53. 10.1002/jcb.21206 17211835

[141] Undas A Brummel-Ziedins KE Mann KG. Statins and blood coagulation. Arterioscler Thromb Vasc Biol. (2005) 25:287–94. 10.1161/01.ATV.0000151647.14923.ec 15569822

[142] Eto M Kozai T Cosentino F Joch H Lüscher TF. Statin prevents tissue factor expression in human endothelial cells: role of Rho/Rho-kinase and Akt pathways. Circulation. (2002) 105:1756–9. 10.1161/01.CIR.0000015465.73933.3B 11956113

[143] Bianconi V Sahebkar A Banach M Pirro M. Statins, haemostatic factors and thrombotic risk. Curr Opin Cardiol. (2017) 32:460–6. 10.1097/HCO.0000000000000397 28266936

[144] Rawish E Sauter M Sauter R Nording H Langer HF. Complement, inflammation and thrombosis. Br J Pharmacol. (2021) 178:2892–904. 10.1111/bph.15476 33817781

[145] Ramcharan AS Van Stralen KJ Snoep JD Mantel-Teeuwisse AK Rosendaal FR Doggen CJ. HMG-CoA reductase inhibitors, other lipid-lowering medication, antiplatelet therapy, and the risk of venous thrombosis. J Thromb Haemost. (2009) 7:514–20. 10.1111/j.1538-7836.2008.03235.x 19036068

[146] Hilgendorff A Muth H Parviz B Staubitz A Haberbosch W Tillmanns H. Statins differ in their ability to block Nf-KappaB activation in human blood monocytes. Int J Clin Pharmacol Ther. (2003) 41:397–401. 10.5414/CPP41397 14518599

[147] Laforge M Elbim C Frere C Hemadi M Massaad C Nuss P. Tissue damage from neutrophil-induced oxidative stress in covid-19. Nat Rev Immunol. (2020) 20:515–6. 10.1038/s41577-020-0407-1 32728221 PMC7388427

[148] Codo AC Davanzo GG Monteiro LD de Souza GF Muraro SP Virgilio-da-Silva JV. Elevated glucose levels favor SARS-CoV-2 infection and monocyte response through a HIF-1 alpha/glycolysis-dependent axis. Cell Metab. (2020) 32:437–6.e5. 10.2139/ssrn.3606770 32697943 PMC7367032

[149] Schönrich G Raftery MJ Samstag Y. Devilishly radical network in covid-19: oxidative stress, neutrophil extracellular traps (Nets), and T cell suppression. Adv Biol Regul. (2020) 77:100741. 10.1016/j.jbior.2020.100741 32773102 PMC7334659

[150] Yang S Shih HJ Chow YC Wang TY Tsai PS Huang CJ. Simvastatin attenuates testicular injury induced by torsion-detorsion. J Urol. (2010) 184:750–6. 10.1016/j.juro.2010.03.103 20639051

[151] Pignatelli P Sanguigni V Lenti L Loffredo L Carnevale R Sorge R. Oxidative stress-mediated platelet Cd40 ligand upregulation in patients with hypercholesterolemia: effect of atorvastatin. J Thromb Haemost. (2007) 5:1170–8. 10.1111/j.1538-7836.2007.02533.x 17388962

[152] Pignatelli P Carnevale R Pastori D Cangemi R Napoleone L Bartimoccia S. Immediate antioxidant and antiplatelet effect of atorvastatin *via* inhibition of Nox2. Circulation. (2012) 126:92–103. 10.1161/CIRCULATIONAHA.112.095554 22615342

[153] Violi F Carnevale R Pastori D Pignatelli P. Antioxidant and antiplatelet effects of atorvastatin by Nox2 inhibition. Trends Cardiovasc Med. (2014) 24:142–8. 10.1016/j.tcm.2013.09.006 24263084

[154] Liu A Wu Q Guo J Ares I Rodriguez JL Martinez-Larranaga MR. Statins: adverse reactions, oxidative stress and metabolic interactions. Pharmacol Ther. (2019) 195:54–84. 10.1016/j.pharmthera.2018.10.004 30321555

[155] Rodrigues-Diez RR Tejera-Muñoz A Marquez-Exposito L Rayego-Mateos S Santos Sanchez L Marchant V. Statins: could an old friend help in the fight against covid-19? Br J Pharmacol. (2020) 177:4873–86. 10.1111/bph.15166 32562276 PMC7323198

[156] Katsiki N Banach M Mikhailidis DP. Lipid-lowering therapy and renin-angiotensin-aldosterone system inhibitors in the era of the covid-19 pandemic. Arch Med Sci. (2020) 16:485–9. 10.5114/aoms.2020.94503 32399093 PMC7212217

[157] Stroes ES Thompson PD Corsini A Vladutiu GD Raal FJ Ray KK. Statin-Associated muscle symptoms: impact on statin therapy-european atherosclerosis society consensus panel statement on assessment, aetiology and management. Eur Heart J. (2015) 36:1012–22. 10.1093/eurheartj/ehv043 25694464 PMC4416140

[158] Nguyen KA Li L Lu D Yazdanparast A Wang L Kreutz RP. A comprehensive review and meta-analysis of risk factors for statin-induced myopathy. Eur J Clin Pharmacol. (2018) 74:1099–109. 10.1007/s00228-018-2482-9 29785580

[159] Disser NP De Micheli AJ Schonk MM Konnaris MA Piacentini AN Edon DL. Musculoskeletal consequences of covid-19. J Bone Joint Surg Am. (2020) 102:1197–204. 10.2106/JBJS.20.00847 32675661 PMC7508274

[160] Rosenson RS Baker S Banach M Borow KM Braun LT Bruckert E. Optimizing cholesterol treatment in patients with muscle complaints. J Am Coll Cardiol. (2017) 70:1290–301. 10.1016/j.jacc.2017.07.752 28859793

[161] Li J Fan JG. Characteristics and mechanism of liver injury in 2019. Coronavirus Disease. J Clin Transl Hepatol. (2020) 8:13–7. 10.14218/JCTH.2020.00019 32274341 PMC7132021

[162] Stefan N Birkenfeld AL Schulze MB. Global pandemics interconnected - obesity, impaired metabolic health and covid-19. Nat Rev Endocrinol. (2021) 17:135–49. 10.1038/s41574-020-00462-1 33479538

[163] Zhang Y Xue W Zhang W Yuan Y Zhu X Wang Q. Histone methyltransferase G9a protects against acute liver injury through Gstp1. Cell Death Differ. (2020) 27:1243–58. 10.1038/s41418-019-0412-8 31515511 PMC7206029

[164] Liu C Wang J Wei Y Zhang W Geng M Yuan Y. Fat-specific knockout of Mecp2 upregulates slpi to reduce obesity by enhancing browning. Diabetes. (2020) 69:35–47. 10.2337/db19-0502 31597640

[165] Chen H Huang Y Zhu X Liu C Yuan Y Su H. Histone demethylase Utx Is a therapeutic target for diabetic kidney disease. J Physiol. (2019) 597:1643–60. 10.1113/JP277367 30516825 PMC6418754

[166] Galicia-Garcia U Jebari S Larrea-Sebal A Uribe KB Siddiqi H Ostolaza H. Statin treatment-induced development of type 2 diabetes: from clinical evidence to mechanistic insights. Int J Mol Sci. (2020) 21:725. 10.3390/ijms21134725 32630698 PMC7369709

[167] Yang C Zhang Y Zeng X Chen H Chen Y Yang D. Kidney injury molecule-1 is a potential receptor for SARS-CoV-2. J Mol Cell Biol. (2021) 13:185–96. 10.1093/jmcb/mjab003 33493263 PMC7928767

[168] Yang D Xiao Y Chen J Chen Y Luo P Liu Q. Covid-19 and chronic renal disease: clinical characteristics and prognosis. QJM. (2020) 113:799–805. 10.1093/qjmed/hcaa258 32840579 PMC7499788

[169] Chen Y Yang D Cheng B Chen J Peng A Yang C. Clinical Characteristics and outcomes of patients with diabetes and covid-19 in association with glucose-lowering medication. Diabetes Care. (2020) 43:1399–407. 10.2337/dc20-0660 32409498

[170] Mach F Ray KK Wiklund O Corsini A Catapano AL Bruckert E. Adverse effects of statin therapy: perception Vs. The evidence - focus on glucose homeostasis, cognitive, renal and hepatic function, haemorrhagic stroke and cataract. Eur Heart J. (2018) 39:2526–39. 10.1093/eurheartj/ehy182 29718253 PMC6047411

[171] Ward NC Watts GF Eckel RH. Statin toxicity. Circ Res. (2019) 124:328–50. 10.1161/CIRCRESAHA.118.312782 30653440

[172] Aghagoli G Gallo Marin B Katchur NJ Chaves-Sell F Asaad WF Murphy SA. Neurological involvement in covid-19 and potential mechanisms: a review. Neurocrit Care. (2021) 34:1062–71. 10.1007/s12028-020-01049-4 32661794 PMC7358290

[173] Mach F Baigent C Catapano AL Koskinas KC Casula M Badimon L. (2019). Esc/Eas guidelines for the management of dyslipidaemias: lipid modification to reduce cardiovascular. Risk Eur Heart J. (2020) 41:111–88. 10.1093/eurheartj/ehz455 31504418

[174] Zhao M Luo Z He H Shen B Liang J Zhang J. Decreased low-density lipoprotein cholesterol level indicates poor prognosis of severe and critical covid-19 patients: a retrospective, single-center study. Front Med. (2021) 8:585851. 10.3389/fmed.2021.585851 34124081 PMC8187559

[175] Wei X Zeng W Su J Wan H Yu X Cao X. hypolipidemia is associated with the severity of covid-19. J Clin Lipidol. (2020) 14:297–304. 10.1016/j.jacl.2020.04.008 32430154 PMC7192140

[176] Fogacci F Borghi C Cicero AFG. Misinterpreting data in lipidology in the era of covid-19. J Clin Lipidol. (2020) 14:543–4. 10.1016/j.jacl.2020.07.004 32768364 PMC7343642

[177] Black S Nicolay U Del Giudice G Rappuoli R. Influence of statins on influenza vaccine response in elderly individuals. J Infect Dis. (2016) 213:1224–8. 10.1093/infdis/jiv456 26516142

[178] Havers FP Chung JR Belongia EA McLean HQ Gaglani M Murthy K. Influenza vaccine effectiveness and statin use among adults in the United States, 2011-2017. Clin Infect Dis. (2019) 68:1616–22. 10.1093/cid/ciy780 30371753 PMC6495015

[179] Menni C Klaser K May A Polidori L Capdevila J Louca P. Vaccine side-effects and SARS-CoV-2 infection after vaccination in users of the covid symptom study app in the Uk: a prospective observational study. Lancet Infect Dis. (2021) 21:939–49. 10.1016/S1473-3099(21)00224-3 33930320 PMC8078878

[180] Watanabe M Balena A Tuccinardi D Tozzi R Risi R Masi D. Central obesity, smoking habit, and hypertension are associated with lower antibody titres in response to Covid-19 mRNA vaccine. Diabetes Metab Res Rev. (2022) 38:e3465. 10.1002/dmrr.3465 33955644 PMC8209952

[181] Wen W Chen C Tang J Wang C Zhou M Cheng Y. Efficacy and safety of three new oral antiviral treatment (molnupiravir, fluvoxamine and paxlovid) for Covid-19: a meta-analysis. Ann Med. (2022) 54:516–23. 10.1080/07853890.2022.2034936 35118917 PMC8820829

[182] Saravolatz LD Depcinski S Sharma M. Molnupiravir and nirmatrelvir-ritonavir: oral covid antiviral drugs. Clin Infect Dis. (2022). 10.1093/cid/ciac180. [Epub ahead of print]. 35245942 PMC9383515

[183] Mahase E. Covid-19: pfizer's paxlovid is 89% effective in patients at risk of serious illness, company reports. BMJ. (2021) 375:n2713. 10.1136/bmj.n2713 34750163

[184] Couzin-Frankel J. Antiviral pills could change pandemic's course. Science. (2021) 374:799–800. 10.1126/science.acx9605 34762459

